# Survival Motor Neuron (SMN) protein is required for normal mouse liver development

**DOI:** 10.1038/srep34635

**Published:** 2016-10-04

**Authors:** Eva Szunyogova, Haiyan Zhou, Gillian K. Maxwell, Rachael A. Powis, Muntoni Francesco, Thomas H. Gillingwater, Simon H. Parson

**Affiliations:** 1 Institute of Medical Sciences, University of Aberdeen, Foresterhill, Aberdeen, United Kingdom; 2 Euan MacDonald Center for Motor Neurone Disease Research, University of Edinburgh, Edinburgh, United Kingdom; 3 Dubowitz Neuromuscular Centre, Institute of Child Health, University College London, London, United Kingdom; 4 Center for Integrative Physiology, University of Edinburgh, Edinburgh, United Kingdom

## Abstract

Spinal Muscular Atrophy (SMA) is caused by mutation or deletion of the survival motor neuron 1 (*SMN1*) gene. Decreased levels of, cell-ubiquitous, SMN protein is associated with a range of systemic pathologies reported in severe patients. Despite high levels of SMN protein in normal liver, there is no comprehensive study of liver pathology in SMA. We describe failed liver development in response to reduced SMN levels, in a mouse model of severe SMA. The SMA liver is dark red, small and has: iron deposition; immature sinusoids congested with blood; persistent erythropoietic elements and increased immature red blood cells; increased and persistent megakaryocytes which release high levels of platelets found as clot-like accumulations in the heart. Myelopoiesis in contrast, was unaffected. Further analysis revealed significant molecular changes in SMA liver, consistent with the morphological findings. Antisense treatment from birth with PMO25, increased lifespan and ameliorated all morphological defects in liver by postnatal day 21. Defects in the liver are evident at birth, prior to motor system pathology, and impair essential liver function in SMA. Liver is a key recipient of SMA therapies, and systemically delivered antisense treatment, completely rescued liver pathology. Liver therefore, represents an important therapeutic target in SMA.

Spinal Muscular Atrophy (SMA) is a mainly childhood-onset form of motor neuron disease^1^,^2^, caused by a deletion or loss-of-function mutation of the *Survival Motor Neuron-1 (SMN1)* gene^3^. The protein product; Survival Motor Neuron (SMN) is a cell-ubiquitous, key component of the spliceosome, and deletion is embryonic lethal^4^. However, the activity of the *SMN2* copy gene in humans, compensates by generating sufficient full-length protein to ensure survival, though the predominate protein produced is truncated due to exon skipping in most organs^4^. The SMN protein was characterised and named according to an apparent, specific vulnerability of motor neurons to low levels of the protein^4^. But, the absolute dependence of all cells on SMN protein has directed us to examine non-motor pathologies in SMA, in order to further understand the role of this multi-functional protein during development.

Reduced SMN protein levels result in a wide range of systemic pathologies including; lung, heart, vascular system^5^,^6^ and testis^7^. These systemic defects must be described and understood before effective therapies can be designed. Significantly, there is considerable evidence of circulatory system defects in animal models and SMA patients, with reports of defects in heart morphology^8^,^9^,^10^,^11^,^12^,^13^,^14^,^15^,^16^,^17^,^18^,^19^ and function^20^,^21^,^22^,^23^,^24^,^25^,^26^. In addition, there is a growing body of evidence to support a wide range of defects affecting the structural and circulating component of the vascular system: capillaries are depleted in muscle and spinal cord^27^,^28^, resulting in significant tissue hypoxia^28^; red and white cell counts are altered^29^, blood clots are present^30^, anti-coagulation therapy resolves digital necrosis^30^,^31^; SMN protein is enriched in anuclear platelets^32^ and platelet numbers are altered by therapeutic interventions in SMA^29^.

The liver has high levels of SMN protein expression^33^ and is a significant player in the production of platelets by resident megakaryocytes at the early stages of development^34^, and therefore a likely target for aberrant blood cell production. However, pathology in the liver is poorly described in SMA, though several lines of research have implicated its involvement: liver has a higher level of SMN expression than spinal cord in adult human tissue^33^ and targeted deletion of SMN in embryonic mouse liver results in dramatic atrophy and severe dysfunction, iron overload, failure to regenerate and late embryonic lethality^35^. Significantly, increasing SMN protein levels in neonatal liver by systemic administration of antisense oligonucleotide (AO-10-27), robustly rescues severe SMA mice^36^, and this peripheral restoration of SMN compensates for its deficiency in the CNS and preserves motor neurons^36^.

Given the requirement for SMN protein in liver, and its implication in downstream disease mechanisms, we have carried out a detailed morphological, molecular and functional assessment of liver in a mouse model of severe SMA. We show delayed development of hepatic plates with an abnormal preservation of foetal morphology and cellular processes into the postnatal period. Specifically, erythropoiesis is prolonged, resulting in congestion of liver sinusoids with erythrocyte precursor cells and shedding of excessive numbers of platelets into the circulatory system, which aggregate into clot-like accumulations. Corroborative molecular changes are present in developmental and erythropoietic pathways, and antisense restoration of SMN from birth, results in full recovery of liver morphology. Finally, we uncovered an unexpected *SMN2* splicing pattern in control liver, suggesting that developmental shifts in splicing may be important in organs other than the testis^7^.

These findings suggest a key dependence on normal SMN protein levels for liver development and function, and that deficiency results in prolonged extramedullary erythropoiesis leading to altered blood composition and function. These data further elucidate the functional complexity of the SMN protein.

## Results

### SMA Liver is Relatively Normal in Form but Has a Distinctive Dark Red Colour

**Figure 1:**
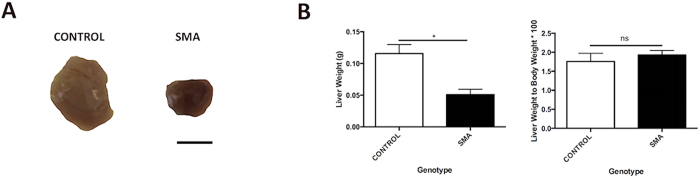
SMA Liver is Relatively Normal in Size with a Distinctive Dark Red Phenotype. (**A**) Gross anatomy of SMA liver harvested from late symptomatic Taiwanese mice. Scale bar, 50 mm. (**B**) Quantification of liver weight of P9 Taiwanese mice. *p* values were calculated using two-tailed Student’s *t*-test. Error bars, mean ± s.e.m. (n = 3 mice per group).

Gross anatomical analysis of SMA liver revealed no detectable structural abnormalities. The SMA liver was significantly smaller when compared to littermate control (*p < 0.05) however its reduced size was in proportion to reduced body weight (ns *p* > 0.05). The most striking difference between SMA liver and its littermate control was the dark-red colouration, which was present at birth (P1) and maintained until a late symptomatic timepoint (P9: Fig. 1A,B).

### SMA Liver Shows Developmental Failure

**Figure 2:**
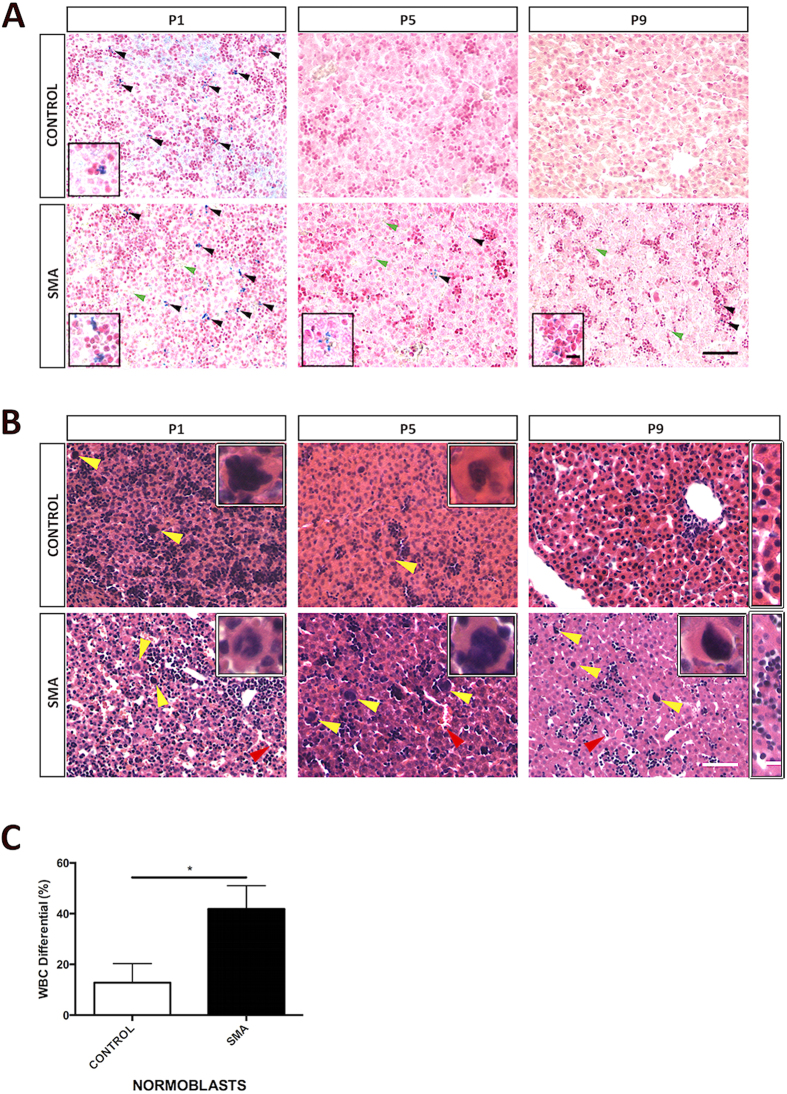
Persistent Erythropoietic Elements in SMA Liver Shows Developmental Failure. (**A**) Representative light microscopy of Perl’s staining of livers at birth (P1) and postnatal days 5 (P5) and 9 (P9). Note the presence of iron deposits (black arrowheads) in later stages of development in SMA liver. P9 SMA liver appears to lack the organised hepatic plate structure as seen in control with predominant erythroblastic islands (dark red nuclei clusters). Green arrowheads point to RBCs outside main vessels. Magnified panels at bottom left show iron deposits (blue). Scale bar, 50 μm. Magnified panel scale bar, 10 μm. (**B**) Representative light microscopy of H&E-stained micrographs of livers. Red arrowheads show nucleated RBC within sinusoid. Yellow arrowheads and magnified panels at top right show megakaryocytes. P9 rectangular magnified panel shows nicely formed hepatic plate in control and lack thereof in SMA. Scale bar, 50 μm. Magnified panel scale bar, 10 μm. (**C**) White blood cell (WBC) differential showing the percentage of normoblasts in control and SMA blood samples obtained from P8 Taiwanese mice. *p* values were calculated using two-tailed Student’s *t*-test. Error bars, mean ± s.e.m. (n = 4 mice per group).

We examined liver function by looking for iron accumulation in SMA liver, as complete loss of SMN results in embryonic iron overload^35^. Perl’s staining revealed small iron deposits at birth (P1) in both control and SMA liver (Fig. 2A). These disappear as development progresses in control, and none are present at P5 or P9. However, in SMA liver iron deposition, indicative of defective iron homeostasis persists through P5 and P9 (Fig. 2A). Closer inspection also revealed the presence of small numbers of disorganised sinusoids and a failure to develop clearly defined sinusoidal spaces between hepatic plates as present in control. In addition, the majority of the tissue is dominated by dense clusters of dark red nuclei, characteristic of sites of blood cell production in erythroblastic islands. Finally, the SMA liver appears to have more red blood cells (RBC) within the sinusoids, but not in the major blood vessels from P1 to P9.

To further examine these structural defects, we carried out H&E histological analysis of liver. This confirmed the presence of poorly developed sinusoids and a relatively immature morphology at all ages examined in SMA (Fig. 2B). Active haematopoiesis, indicated by the presence of haematopoietic elements: erythroblastic islands, megakaryocytes and granulocytes is present at birth (P1) in both control and SMA liver. As development proceeds, these haematopoietic elements decrease and eventually disappear, while well-organised and differentiated, one to two hepatocytes wide, mature hepatic plates form in the control (Fig. 2B). However, in the SMA liver active haematopoiesis is maintained, evident by the high number of haematopoietic elements present (Fig. 2B). In addition, wide sinusoids indicating a failure of the liver parenchyma to expand are present at birth (Fig. 2B). Hepatocytes remain disorganised and do not mature into hepatic plates in SMA liver (Fig. 2B. Histology indicated that some of the RBCs within SMA liver sinusoids appear to be nucleated, which was confirmed upon further blood analysis (Fig. 2C), as SMA mice have significantly increased number of normoblasts, immediate precursors of RBCs, (*p < 0.05) at P8.

Taken together, the findings of iron accumulation, persistent haematopoietic elements and delayed morphological maturation, suggest developmental failure in SMA liver.

### Erythropoiesis is Elevated and Prolonged in SMA Liver

**Figure 3:**
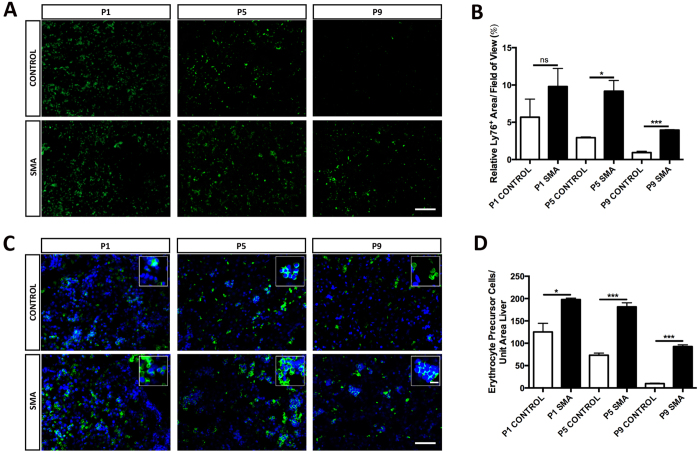
Prolonged Active Erythropoiesis in SMA Liver. (**A**) Representative micrographs labelled with the Ly76 marker (green), in sections of liver obtained from Taiwanese mice. Scale bar, 100 μm. (**B**) Quantification of erythrocytes and their precursors expressed as a percentage of cross-sectional liver area. *p* values were calculated using two-tailed Student’s *t*-test. Error bars, mean ± s.e.m. (n ≥ 3 mice per group). (**C**) Representative micrographs and their magnified panels co-stained with the Ly76 marker (green) and DAPI (blue), in sections of liver obtained from Taiwanese mice. Scale bar, 50 μm. Magnified panel scale bar, 10 μm. (**D**) Quantification of erythrocyte precursor cells expressed as a percentage of cross-sectional liver area. *p* values were calculated using two-tailed Student’s *t*-test. Error bars, mean ± s.e.m. (n ≥ 3 mice per group).

The SMA liver is dark red, developmentally immature and congested with blood. We first confirmed the presence of abnormal quantities of RBC in SMA liver sinusoids by staining with Ly76 for RBC and RBC precursors (Fig. 3A,B). In control liver, the amount of Ly76 positive RBC and precursors is initially low and rapidly decreased as development progressed, resulting in the presence of only a few, scattered Ly76-positive cells at P9 (Fig. 3A). SMA liver showed elevated levels at birth (P1: non-significant), which were maintained (P5: *p < 0.05) began to fall, but remained 4 times greater than controls (P9: ***p < 0.001) (Fig. 3B). Further analysis, by co-staining with a nuclear marker DAPI, revealed these cells to be nucleated and therefore erythrocyte precursor cells (Fig. 3C,D), which were elevated at birth (P1: *p < 0.05) remained high at (P5: ***p < 0.001), ultimately decreased (P9: ***p < 0.001) but remained almost 10 times greater than in control.

### Megakaryocytes Persist and Produce Abnormally High Levels of Platelets in SMA Liver

**Figure 4:**
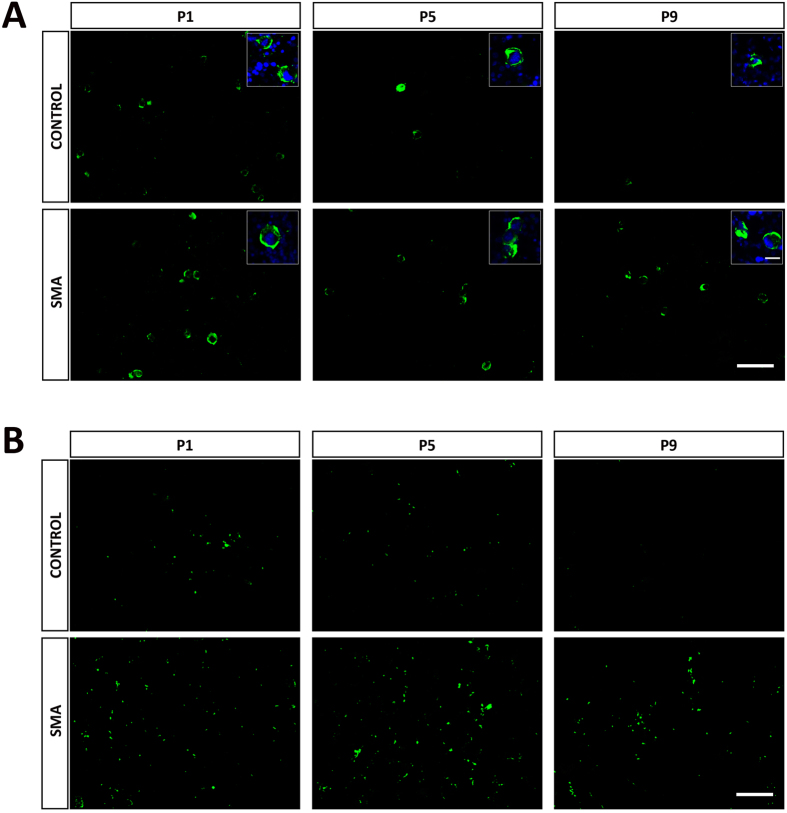
Megakaryocytes Persist and Produce Abnormally High Levels of Platelets in SMA Liver. (**A**) Representative micrographs of CD41 (green) staining for erythroid lineage and their magnified panels co-stained with DAPI (blue), showing megakaryocytes in sections of liver from Taiwanese mice. Scale bar, 100 μm. Magnified panel scale bar, 10 μm. (**B**) Representative micrographs of CD41 (green) staining showing platelets in sections of liver from Taiwanese mice. Scale bar, 50 μm.

Our histological findings indicated increased numbers of megakaryocytes in the SMA liver, and with the evidence of prolonged erythropoiesis, we next sought to determine if megakaryopoiesis was also affected. First, we stained sections with CD41, a well-characterised marker for megakaryocytes (Fig. 4A). Megakaryocytes are generally sparse, and the low levels present at birth, slowly decreased to near zero in control liver by P9 (Fig. 4A). In SMA liver there appear to be more megakaryocytes present at birth, and only a slight decline in numbers through P5 to P9 (Fig. 4A).

CD41 also identifies platelets, apparent as clumps of much smaller structures, which are easily differentiated from the megakaryocytes by size and absence of nuclei (Fig. 4B). These can be used as a surrogate marker for megakaryopoiesis and in control liver, platelet numbers were low at birth and P5, falling to almost zero by P9 (Fig. 4B). The situation was quite different in SMA liver, where increased numbers of platelets was present at birth, and maintained through P5 to P9 (Fig. 4B). This confirms abnormal megakaryopoiesis at all developmental stages in SMA liver.

### Platelets Aggregate into Circulating Clot-like Accumulations in SMA

**Figure 5:**
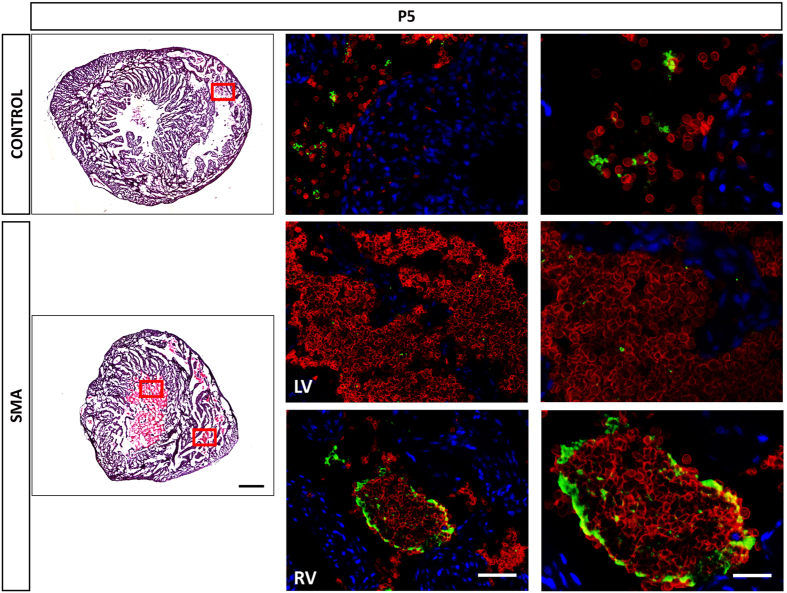
Platelets Aggregate into Circulating Clot-like Accumulations in SMA. Representative micrographs of H&E and RBCs (Ly76^+^ DAPI^−^ stain - red) and platelets (CD41^+^ stain - green) co-stained with DAPI (blue) of heart sections obtained at P5 from Taiwanese mice. Red squares show where Immunofluorescence images were taken. LV = Left Ventricle. RV = Right Ventricle. Note that the blood accumulation observed in SMA LV is not clotted unlike the one in RV. Scale bar for H&E, 500 μm. Scale bar for Immunofluorescence - left, 50 μm and - right, 10 μm.

We were keen to determine if this increase in platelet production and retention by liver results in changes in circulating platelets. We therefore investigated the heart from the same animals that the liver was obtained to look for any evidence of abnormalities in circulating platelets. Initial histological assessment immediately indicated substantial accumulation of blood in both ventricles of SMA heart, which was not present in littermate controls (Fig. 5). We co-stained sections of heart at P5 with CD41 for megakaryocytes and platelets, Ly76 for the RBC lineage and DAPI for nuclei to differentiate mature RBC from their nucleated precursors. At P5, large accumulations of mature RBC are present in both ventricles, but closer inspection shows those in the right ventricle include significant accumulations of platelets (Fig. 5). The combined RBC and platelet aggregates have the appearance of a clot. Interestingly, the blood in left ventricle of SMA mice is different, and does not exhibit clot-like appearance (Fig. 5), but is not observed in control littermates and suggests gross changes in circulating blood composition and/or cardiac dysfunction. These data suggest that the prolonged and abnormal production of platelets and RBC precursors by the liver, leads to changes in circulating blood composition and formation of clot-like accumulations.

### Myelopoiesis Does Not Appear to Be Affected in SMA Liver

**Figure 6:**
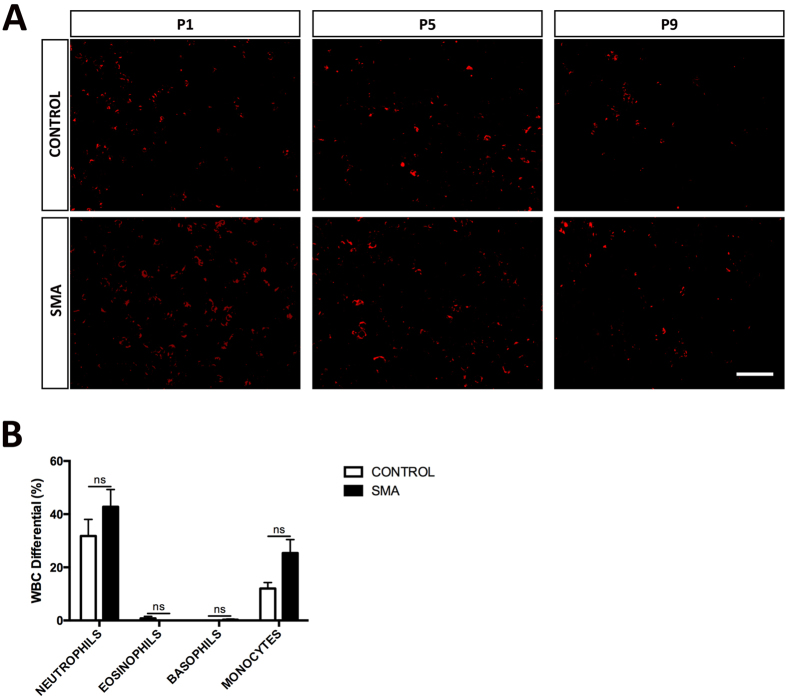
Myelopoiesis Does Not Appear to Be Affected in SMA Liver. (**A**) Representative micrographs labelled with the CD11b to show myeloid leukocytes (red), in sections of liver obtained from Taiwanese mice. Scale bar, 50 μm. (**B**) WBC differential showing the percentage of granulocytes and monocytes in control and SMA blood samples obtained from P8 Taiwanese mice. *p* values were calculated using two-tailed Student’s *t*-test. Error bars, mean ± s.e.m. (n = 4 mice per group).

Finally, to investigate if developmental failure was a general feature of SMA liver, we stained sections with CD11b, an integrin expressed on the surface of myeloid leukocytes, to identify cells of the myelopoiesis lineage, a significant pathway normally also activated in haematopoiesis. Our staining revealed no apparent differences in the presence of granulocytes and monocytes in control and SMA liver, and a predicted decrease in their levels as development progressed (Fig. 6A). This suggests that the generation of white blood cells (myelopoiesis) is not defective in SMA liver. Results from a white blood cell (WBC) differential analysis (Fig. 6B) showed no significant differences in the numbers of circulating myeloid leukocytes (neutrophils, eosinophils, basophils and monocytes) between SMA and control at late symptomatic age (P8).

These data suggest that developmental failure is not a generalised feature of liver development in SMA, but rather specific pathways are selectively targeted by low levels of SMN protein.

### Molecular Pathways are Modified in SMA Liver

**Figure 7:**
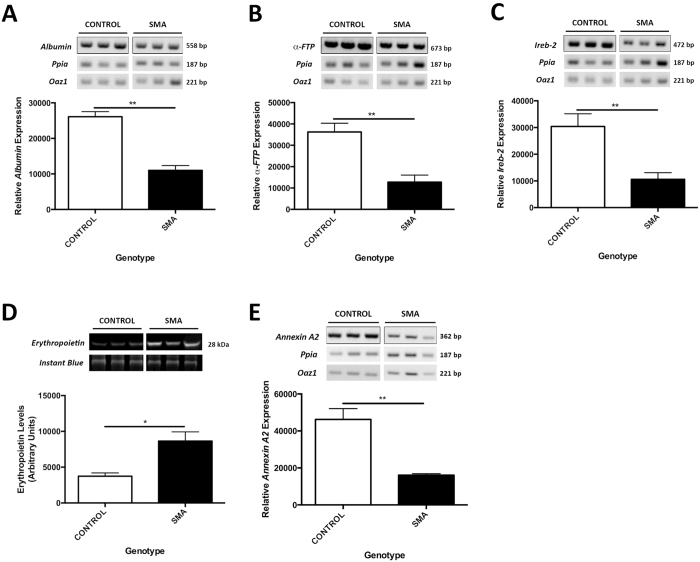
Molecular Pathways are Modified in SMA Liver. Semi-quantitative RT-PCR analysis of *albumin* (**A**), *α-FTP* (**B**), *Ireb-2* (**C**) and *Annexin A2* (**E**) transcripts in control and SMA livers normalised to *Ppia* and *Oaz1*. (**D**) Total erythropoietin protein levels analysed by Western Blot and normalised to the total protein (Instant Blue). *p* values were calculated using two-tailed Student’s *t*-test. Error bars, mean ± s.e.m. (n ≥ 3 mice per group). For uncropped gels/blot see Supplementary Figure 1.

In order to investigate which pathways were affected by low SMN levels, we carried out preliminary molecular analysis by RT-PCR and western blot. Using RT-PCR, we show a dramatic reduction in both *albumin* (Fig. 7A: **p < 0.01) and *α-FTP* (Fig. 7B: **p < 0.01) mRNA expression in SMA liver compared to control at a late symptomatic time point (P11). These results support our detailed histological assessment of the liver showing failed development as a key general finding. Importantly, these molecules were also identified following liver-specific knockout of SMN^35^.

We also found an increase in iron accumulation, indicated by histological Perl’s stain (Fig. 2A), in SMA liver. Therefore, we next assessed the expression of *Ireb-2*, which is reduced in SMN-depleted embryonic stem cells^38^, and important in cellular iron homeostasis^37^. *Ireb-2* mRNA expression was also significantly depleted in SMA liver (Fig. 7C: **p < 0.01) at a late symptomatic time point (P11).

In addition, SMA liver was dark red in colour and congested with blood. Significantly many of the red blood cells were immature, nucleated normoblasts, indicative of ongoing erythropoiesis. Therefore, we next assessed erythropoetin (EPO), the master regulator of erythropoiesis^39^. We found a significant increase in EPO protein in SMA liver at a mid/late symptomatic time point (P8: Fig. 7D: *p < 0.05).

Finally, a combination of increased megakaryocytes and clot-like accumulations of platelets in SMA liver and heart, prompted us to investigate *Annexin-A2*, an anticoagulant^40^ involved in cellular growth^41^, both of which are affected in SMA liver. We found that *Annexin-A2* mRNA was significantly reduced in SMA liver at a late symptomatic time point (P11: Fig. 7E: **p < 0.01). Interestingly, *Annexin-2* is a binding partner of SMN^42^ and altered in SMA^43^.

Taken together, these data suggest that low levels of SMN are correlated with an altered molecular signature, compatible with the cellular and gross anatomical defects observed in SMA liver.

### Antisense Treatment Prolonged Life and Normalised Liver Development in SMA

Morpholino antisense oligonucleotide treatment, designed to increase SMN protein levels, delivered at birth, specifically rescues a range of neuromuscular pathologies and significantly increases lifespan in SMA mouse models^44^. In addition it also increases SMN levels in non-neuromuscular tissues such as the intestines^45^. We used an identical treatment regime to test the ability of systemically delivered antisense to ameliorate SMA liver pathology.

**Figure 8:**
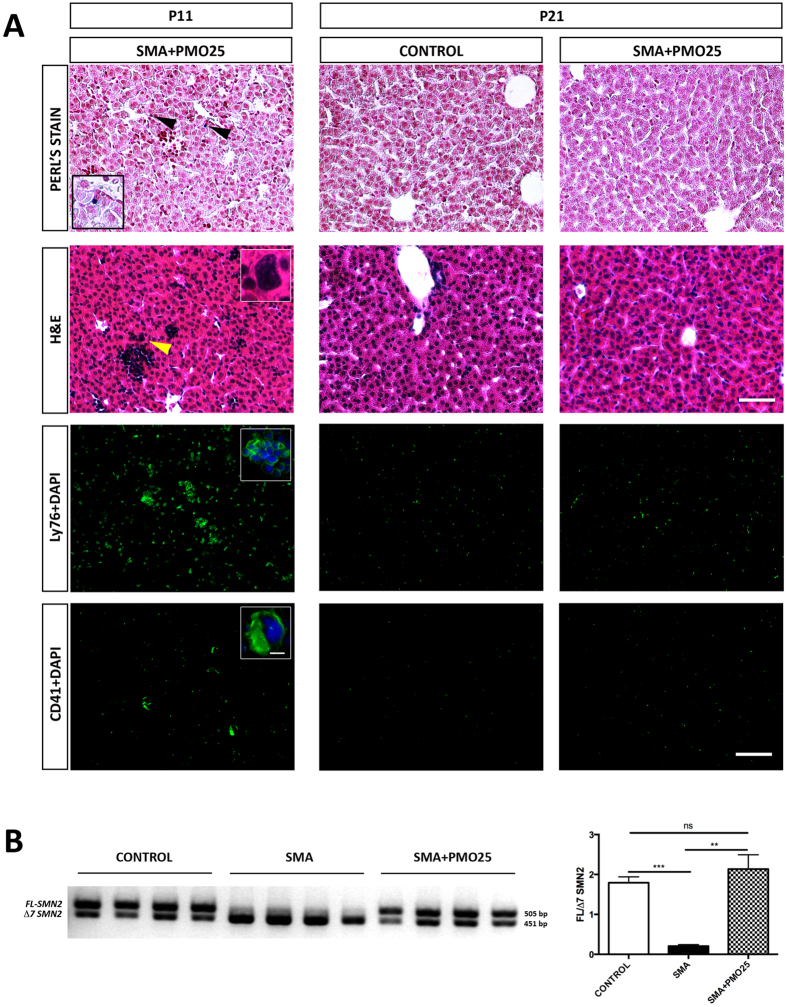
Antisense Treatment Prolonged Life and Normalised Liver Development in SMA. (**A**) Representative micrographs of Perl’s, H&E, Ly76, and CD41 staining of liver obtained from treated and non-treated mice at two different time-points, P11 and P21. Note the persisting presence of iron deposits (black arrowheads, magnified panel), predominant erythroblastic islands, and megakaryocytes in SMA treated liver at P11. This pathology appears to be completely ameliorated at P21. Scale bar: Perl’s and H&E staining, 50 μm; Ly76 and CD41 staining, 100 μm; Magnified panel, 10 μm. (B) Semi-quantitative RT-PCR analysis of the ratio of *FL-SMN2* to *Δ7 SMN2* transcripts between Control, SMA and antisense treated SMA liver at P11. *p* values were calculated using two-tailed Student’s *t*-test. Error bars, mean ± s.e.m. (n = 4 mice per group). For uncropped gel see Supplementary Figure 2.

At late symptomatic age (P11), the SMA-treated liver exhibits no marked improvement compared to the non-treated SMA liver (Fig. 8A). The architecture remains immature, with persistent iron deposits, erythroblastic islands and platelet-producing megakaryocytes (Fig. 8A). However, as the PMO25 treated mice survive for extended periods, we examined the treated liver at P21, and found all aspects of liver pathology to be completely ameliorated. The architecture of PMO25-treated liver at P21 is indistinguishable from an age-matched control liver. There is no evidence of iron deposits, nucleated RBCs or megakaryocytes at P21 (Fig. 8A). These data suggest that the defects in liver development described are specifically due to SMN protein depletion, and can be rescued by its restoration. It is intriguing that rescue of SMA liver pathology, was not complete until beyond the normal lifespan of a severe SMA mouse.

We confirmed that PMO25 antisense treatment was effective in the liver, by specifically looking for increased exon7 inclusion *in SMN2 transcripts*. We found a significant increase in the amount of full-length (including exon7) *SMN2* (*FL-SMN2*) transcript in mid/late symptomatic liver treated with PMO25 antisense at P1 (Fig. 8B: **p < 0.01). Surprisingly, we also found that *FL-SMN2* was the predominant transcript in control liver.

## Discussion

In this study we show that SMN protein is required for normal erythropoiesis and megakaryopoiesis in the liver, with SMN depletion leading to significant defects in liver development in SMA. These defects are corroborated by changes in key pathways including: decreased expression of *albumin* and *α-FTP* mRNAs indicative of a liver-specific developmental defect; decreased expression of *Ireb-2* mRNA correlated with dysregulation of iron homeostasis; elevated levels of EPO protein correlated with prolonged active erythropoiesis and megakaryopoiesis, and a decrease in *Annexin-A2* mRNA expression, an anti-coagulant and known SMN binding partner, which is altered in SMA. Furthermore, we show that antisense treatment increases SMN levels, normalises liver pathology and rescues the mice. Finally, we show that Fl-SMN2 is the predominant SMN transcript in control liver. This adds to the body of evidence suggesting that this antisense treatment regimen offers a promising therapy for patients.

The SMA liver is small but in proportion to lower body mass compared to littermate controls, but exhibits structural abnormalities consistent with developmental failure. Here a reduction in global SMN levels results in iron accumulation and evidence of liver overload at P5 and P9 in SMA. This suggests that liver development is SMN dose-dependent, as deletion of SMN exclusively in the liver results in massive iron overload^35^. Iron accumulation is a sign of immaturity, which is reinforced by our observation of a failure of hepatic plates to mature correctly and prolonged postnatal haematopoiesis. During normal embryonic development, the liver serves as the main haematopoietic organ^46^, peaking at embryonic day 13–14^47^. Over the first 2–4 postnatal days, this role is lost as the bone marrow takes over and this functional shift is reflected in a rapid change in liver architecture to a mature pattern. Our results show that the SMA liver fails to undergo this maturational shift, as haematopoietic elements are still common at P9. This is further supported by the presence of wide, embryonic pattern, sinusoids^48^ filled with normoblasts (nucleated RBC) in SMA.

Complete deletion of the SMN protein specifically targeted to liver resulted in severe impairment of liver development and embryonic lethality^35^. They suggested that functional defects within the liver are likely due to an imbalance between haematopoietic elements and maturing hepatocytes. This is consistent with reduction of *albumin* and *α-FTP* mRNA expression, both of which are almost exclusively expressed in the liver, predominantly by the hepatocytes^49^, and are decreased following liver-specific knockout of SMN. This is indicative of a liver-specific dysfunction associated with marked reduction in the presence of mature, functional hepatocytes. The recent availability of induced pluripotent stem cells generated from SMA human fibroblasts^50^, now provides the opportunity to map the developmental changes in hepatocytes in SMA, and potentially identify direct links between decrease SMN and defects in liver development in the future.

The increase in RBC and platelets in combination with this decreased expression of *Annexin*-*A2* mRNA, likely contributes to the clot-like accumulations found in the circulatory system. We speculate that these may be associated with commonly reported cardiac defects^16^,^17^,^18^,^19^,^20^,^21^,^22^,^23^,^30^,^31^ and distal necrosis^30^,^31^ reported in SMA patients.

The pronounced dark-red phenotype in SMA liver is consistent with the observed increase in RBC as a result of on-going erythropoiesis and iron overload, which is necessary for erythroblast maturation^51^. Therefore, iron accumulation and accompanying decrease in *Ireb-2* mRNA expression in SMA liver are a likely outcome of persistent active erythropoiesis at later stages of development. *Ireb-2*, which is a key regulator of vertebrate cellular iron homeostasis^37^, is significantly reduced in embryonic stem cells with low SMN protein^38^. Further, motor neurons from *Ireb-2*^−/−^ mice display increased ferritin and impaired mitochondrial function resulting in motor neuron degeneration^52^. We show increased hepatic iron and significant a reduction in *Ireb-2 mRNA* in SMA liver, which taken together with mitochondrial dysfunction in SMA^53^,^54^,^55^, suggests that iron dysregulation may be important in motor neuron degeneration, and a candidate for further study in SMA.

The increase in megakaryocytes and platelets in SMA liver indicate increased megakaryopoiesis. The elevated levels of platelets may be exacerbated by defects in spleen, as in severe SMA mice the spleen is significantly reduced in size and atrophic^56^,^57^ and contains increased numbers of megakaryocytes^57^. The spleen is the major site for removal of platelets^58^, and a combination of over production and improper removal could result in elevated levels of circulating platelets in SMA.

This in combination with thrombocytosis is predicted to result in clinically-relevant pathologies in SMA patients and animal models. Distal necrosis is a common but unexplained phenotypic feature in both mild and severe SMA mouse models^59^,^60^,^61^,^62^. Further, though infrequent, digital necrosis is observed in infants with severe SMA^30^,^31^, and here a treatment composed mainly of anticoagulants resulted in complete resolution of all lesions^31^. Coagulation studies have not revealed abnormalities in SMA type I patients, but these were likely performed on platelet-free plasma, focusing more on the coagulation factors rather than platelets themselves. The reports of vascular thrombosis and digital necrosis argue that these are not a result of a perfusion abnormality associated with heart defects^30^. Therefore, thrombosis due to increased levels of circulating platelets and decreased *Annexin-A2* may be an important pathological finding in patients. Intriguingly, recent reports indicate that platelets contain surprisingly high amounts of SMN protein^32^, and *Annexin-A2* deficient mice exhibit a hypofibrinolytic phenotype with incomplete clearance of thrombi^63^, suggesting a potential pathway for the promotion of clot formation.

The right atrium of the heart is immediately downstream of the liver, and the presence of clot-like structures in the right ventricle is consistent with the liver as the source of platelets. Extending this idea, we predict blood clots in the lungs, and previous studies have shown dark reddish discoloration in the lungs of SMA mice upon autopsy^62^, a likely indicator of clotting. The presence of fewer clots in the left ventricle of the heart is consistent with a degree of lung-filtering of clots.

We did however find excessive blood pooling in the left ventricle, evidenced by the presence of large numbers of RBC. This could be a result of an increase in total blood volume, bradycardia, or an increase in afterload. The increased production of RBC and platelets described here will increase blood volume and haematocrit. Such an increase in blood viscosity will put additional stress on the heart, which could explain our observation of left ventricular blood accumulation and also of cardiomyopathy in SMA patients^16^,^17^,^30^,^31^ and animal models of SMA^18^,^19^,^24^,^25^,^26^. Severe bradycardia is described in many type I patients^20^,^22^, which might also be associated with blood pooling in the left ventricle. Finally, the reduction in capillary bed density in patient muscle biopsies^28^ and the muscle and spinal cord of SMA mice^27^,^28^ would increase afterload leading to blood pooling within the left ventricle.

Our observation that myelopoiesis, shown by unchanged myeloid leukocyte levels, is normal in SMA mice, further strengthens our argument that SMA liver pathology is not the result of generalised development delay, but rather a specific defect. *Annexin-A2* knockdown was shown to negatively influence cell cycling^41^. It is possible that decreased SMN acting via *Annexin-A2* would affect both erythroid and megakaryocytic lineages, as they arise from a common precursor^64^,^65^. Cell cycling defects have been implicated in several studies in SMA: ASO-induced SMN depletion in adult mice showed that cell signalling pathways were most affected implicating SMN in DNA replication and possibly DNA repair^66^; brain weight and cell number changes in mouse model of severe SMA indicated a potential role for SMN in proliferation rather than apoptotic cell death^67^; and *Drosophila* loss or gain of SMN function disrupts larval growth and germline stem cell proliferation and differentiation^68^.

We have shown that systemic treatment with PMO25 antisense increases SMN levels and ameliorates histopathological abnormalities in SMA liver. However, unlike the intestines^45^ where improvement was seen at P11, rescue was delayed in PMO25 treated liver. These data suggest that careful planning of potential combinatorial approaches to SMA therapy will be required to ensure optimal outcomes.

Most interestingly we show that *SMN2* splicing produces more *FL-SMN2* than Δ7SMN2 transcripts in control liver at P11, which challenges the accepted.

SMN2 splicing dogma^3^. These data agree with those in the testis^7^ and indicate a potential splicing switch that requires further investigation.

In conclusion, our data support the view that the reduction in SMN protein levels in severe SMA, results in specific and significant defects in liver development, apparent from birth. These defects in erythropoiesis and megakaryopoiesis but not myelopoiesis, result in potentially wide-ranging systemic pathology through the formation of circulating blood clots, which have been described in both animal models and SMA patients. Our preliminary analysis shows changes in pathways consistent with the developmental defects in liver. We suggest a proteomic approach to not only validate our findings but to also further understanding of the molecular cascades linking SMN-depletion to these specific pathologies, and thereby develop therapies for all aspects of SMA.

## Methods

### Animals

Mouse model used in the following experiments is the Taiwanese, also known as Hsieh-Li mouse model of severe SMA^59^. Taiwanese SMA mice on a congenic FVB background were maintained as breeding pairs (*Smn*^+/−^*x Smn*^−/−^*; SMN2*^*tg*/*tg*^) under standard specific pathogen-free conditions in animal care facilities in Edinburgh or University College London.

All experimental protocols were approved by Edinburgh University research and ethics committees and carried out in accordance with a license from the United Kingdom Home Office under the Animals (Scientific Procedures) Act 1986. Offspring were either homozygous knockouts for *Smn*, *Smn*^−/−^*; SMN2*^*tg*/*0*^, (SMA) or heterozygous, *Smn*^+/−^; *SMN2*^*tg*/*0*^ (Control). Mice were retrospectively genotyped following standard Polymerase Chain Reaction (PCR) protocols^69^. Day of birth was defined as P1.

### Tissue Processing, H&E and Perl’s Staining

All tissue used in these experiments was harvested from mice sacrificed at desired age by intraperitoneal injection of sodium pentobarbital, in accordance with UK the guidance and rules for the use of animals in research and were carried out at the University of Edinburgh. Whole liver and heart were rapidly dissected and fixed for 4 h in 4% paraformaldehyde (PFA). Sequentially, one part of the liver and whole heart were then cryoprotected in 30% sucrose for subsequent OCT embedding, and the other liver part submerged in 70% ethanol to be processed in wax. After sectioning (5 μm), wax embedded liver and OCT embedded heart were stained in Haematoxylin and Eosin (H&E) or for iron (Prussian blue reaction – Mallory’s method) co-labeled with 0.1% Nuclear fast red solution (Sigma-Aldrich N3020) following a standard protocol.

### White Blood Cell Differential

Under terminal anaesthesia induced by isoflurane, the thoracic cavity of Taiwanese SMA mice and their littermates was surgically opened. Using a 30gauge 0.3 ml insulin syringe a blood withdrawn performed by cardiac puncture into the left ventricle. One drop of blood was used to make a blood smear and the remaining placed in a tube with 0.5 M EDTA as an anticoagulant (1:20 anticoagulant to blood ratio) and inverted to ensure proper mix. Mice were culled by exsanguination (non-Schedule 1 procedure). Samples were placed on ice and immediately taken for WBC differential analysis at the Veterinary Pathology Unit, University of Edinburgh.

### Immunohistochemistry

Cryosections were cut at a thickness of 7 μm and stained with rat monoclonal anti-Ly76 primary antibody (Abcam ab91113, 1:100) or rat monoclonal anti-CD41 (AbD Serotec MCA2245GA, 1:100) or rabbit monoclonal anti-CD11b (Abcam ab133357, 1:100). Stains were visualised with Alexa Fluor 488 goat anti-Rat IgG (H + L) (Life Technologies A-11006, 1:200) and Cy3 goat anti-Rat IgG (H + L) (Life Technologies A-10522, 1:200) or Cy3 goat anti-Rabbit IgG (H + L) (Life Technologies A-10520, 1:250). For triple stain (Ly76 co-label with CD41 and DAPI) the staining was carried out subsequently as the primary antibodies were raised in the same host with Ly76 staining performed first. All slides were mounted in Vectashield mounting media containing DAPI (Vector Laboratories H-1200). All sections were imaged using a Nikon eclipse e400 microscope (×10 objective) and its images captured using QICAM Fast 1394 camera and Improvision Velocity 4 image capture software.

### Ly76 Density Imaging and Quantification

The density of Ly76 positive cells in liver was calculated from 3 sections per slide from a minimum of 6 non-consecutive slides for each mouse. Tissues that were poorly stained and/or damaged due to sectioning or where <50% of the area was not occupied by nuclei, due to the presence of large blood vessels were excluded from the analysis. One image approximately in the middle of the whole tissue per section was captured at x100 magnification, and all images were captured at the same exposure. All images sere enhanced in Abode Photoshop (CS4) for contrast and brightness. Using Image J (NIH) the enhanced images were converted to binary where all Ly76 positive cells were assigned black and background white. This allowed calculation of a ratio of black to total white pixels producing a value for relative number of Ly76 positive cells per field of view, expressed as percentage of field of view occupied by relative number of Ly76 positive cells. To eliminate bias, quantification P1 and P9 was carried out on blinded slides.

### Erythrocyte Precursor Cell Imaging and Quantification

For analysis of Ly76 + DAPI positive cells, at least 24 images per liver were analysed. All sections were captured at x200 magnification, enhanced in Abode Photoshop for contrast and brightness prior to merging Ly76-green and DAPI-blue channels. Cells that were positive for Ly76 and DAPI were classified as erythrocyte precursor cells and counted. For cell counting, a random systematic sampling method was used. The random element was created by the unknown orientation of the tissue during its embedding in the OCT. Systematic because imaging of all the livers followed the same rules: (a) four, non-overlapping, images were taken per section starting from the top, left of each section, (b) poorly stained and/or damaged sections were not imaged, (c) images where <50% of the area was not occupied by nuclei, due to the presence of large blood vessels were not imaged, (d) at least six sections per mouse were imaged, and (e) all imaged sections were at least 30 μm apart. To eliminate bias, the quantification of P1 and P9 was carried out on blinded slides.

### AON Treatment of SMA Mice

All AON experimental procedures were performed on mice bred at University College London.

In this study we used a 25-mer morpholino antisense oligomer (PMO25), which targets the ISS-N1^70^, increasing the inclusion of exon7 in *SMN2* transcripts and consequently elevating the total full-length SMN protein levels^36^,^44^,^45^,^71^,^72^,^73^.

PMO25 was synthesised by Gene Tools (Philomath, OR) for research use only. SMA mice were injected with a single subcutaneous dose of 40 μg/g PMO25 at birth. Subcutaneous administrations were injected into the upper part of the back using a 10 μl glass capillary (Drummond Scientific Company #5-000-1001-X10). All treated SMA mice are henceforth referred to as SMA + PMO25.

### Semi-Quantitative Reverse Transcription PCR (RT-PCR)

Extraction of total RNA from liver samples was achieved using RNeasy Mini Kit (Qiagen 74104). RNA concentration was determined by a Nanodrop 2000 spectrophotometer. Total RNA (1 μg) was used for first-strand cDNA synthesis, using SuperScript II Reverse Transcriptase kit (Life Technologies 18064014). The following murine primers were designed for the RT-PCR analysis: *Albumin* (forward, 5′-CCTGCCACCATTTGAAAGGC-3′; reverse, 5′-GTGTCATGCTCCACCTCACT-3′), *α-FTP* (forward, 5′-CTGCTACATTTCGCTGCGTC-3′; reverse, 5′-TGGTTGTTGCCTGGAGGTTT-3′), *Annexin A2* (forward, 5′-CAACCAGGAGCTGCAAGAGA-3′; reverse, 5′-GTGCCTTCTGGTAGTCACCC-3′), *Ireb-2* (forward, 5′-AGCTCCAGACTCCGTGC-3′; reverse, 5′-GGCAGCCCAATCTCTTGAAT-3′), *Ppia*^74^, and *Oaz1*^74^. For *SMN2* transcript analysis human SMN-specific primers were used^44^. Their specificity was tested on a wild type mouse showing no cross-reactivity with the murine *Smn* transcripts (data not shown). Semi-quantification of band intensity was analysed by ImageJ.

### Quantitative Fluorescent Western Blot Analysis

Western blot analysis was performed as previously described^75^. Briefly, liver was manually homogenised and all protein extracted in RIPA buffer (Thermo Scientific™ 89900) containing 1% Halt™ Protease Inhibitor Cocktail (Thermo Scientific™ 78430). Protein concentration was determined by BCA assay (Thermo Scientific™ 23235). Each lane was loaded with 15 μg of protein. Extracted protein was separated by electrophoresis on precast NuPAGE™ Novex™ 4–12% Bis-Tris protein gels (Invitrogen NP0336PK2) and then transferred to nitrocellulose membrane (Novex™ IB23002) using the iBlot 7 minute semi-dry blotting system. Membrane was blocked in Odyssey blocking buffer (LI-COR 927-40000) for 90 minutes at room temperature. Primary antibody against Erythropoietin (Abcam ab129452, 1:200) was diluted in blocking solution and left overnight at 4 °C. Following 6 × 10 minute washes in 0.1M PBS, Alexa Fluor 680 donkey anti-Rabbit Ig (H + L) (Abcam ab186692, 1:10000) was added and membrane incubated for 90 minutes at room temperature. Blots were washed 6 × 10 minutes in 0.1 M PBS and subsequently imaged using an Odyssey Infrared Imaging System and quantified using Image Studio Software (Image Studio^TM^ Lite, Lincoln, NE). Due to alteration in the expression levels of many standard loading control proteins in SMA tissues, total protein levels were used as a loading control^76^. Total protein levels were determined by incubation of control gel in Instant Blue (Sigma-Aldrich ISB1L). Erythropoietin band intensities were then normalised to a loading control to determine final relative protein expression.

### Statistics

All experimental groups consisted of a minimum of 3 different animals, which we have previously shown to be sufficient to attain statistical significance^27^,^28^. Data was collected in Microsoft Excel software (Microsoft Corporation, Redmond, WA). Graphs were generated and statistical analysis performed in GraphPad Prism software (GraphPad Sofware Inc., La Jolla, CA). Bar charts are shown as mean ± SEM. For statistical analysis, unpaired two-tailed Student’s *t*-test has been used and P < 0.05 was considered statistically significant for all analyses.

## Additional Information

**How to cite this article**: Szunyogova, E. *et al*. Survival Motor Neuron (SMN) protein is required for normal mouse liver development. *Sci. Rep*. **6**, 34635; doi: 10.1038/srep34635 (2016).

## Supplementary Material

Supplementary Information
