# Corrigendum: Survival Motor Neuron (SMN) protein is required for normal mouse liver development

**DOI:** 10.1038/srep35898

**Published:** 2016-11-10

**Authors:** Eva Szunyogova, Haiyan Zhou, Gillian K. Maxwell, Rachael A. Powis, Francesco Muntoni, Thomas H. Gillingwater, Simon H. Parson

*Scientific Reports* 6: Article number: 3463510.1038/srep34635; published online: 10 04 2016; updated: 11 10 2016.

The original version of this Article contained errors in the spelling of the author Francesco Muntoni, which was incorrectly given as Muntoni Francesco.

In addition the abbreviation for Rachel A. Powis was incorrectly given as P.A.R. in the Author Contributions statement of the original article.

The Author Contributions statement,

“S.H.P., T.H.G., M.F. and E.S. conceived the project. E.S., H.Z., G.K.M. and P.A.R. carried out all experiments and analysed the data. S.H.P., T.H.G. and E.S. wrote the paper”.

now reads:

“S.H.P., T.H.G., F.M. and E.S. conceived the project. E.S., H.Z., G.K.M. and R.A.P. carried out all experiments and analysed the data. S.H.P., T.H.G. and E.S. wrote the paper”.

These errors have now been corrected in the PDF and HTML versions of the Article.

