# Supplementary material for: Survival Motor Neuron (SMN) protein is required for normal mouse liver development
Source: Sci Rep. 2016 Oct 4;6:34635. doi: 10.1038/srep34635 (PMC5048144; doi:10.1038/srep34635)
Supplement: Supplementary Information [file srep34635-s1.pdf]

Survival Motor Neuron (SMN) protein is required for normal mouse liver development

Supplementary Figures

Szunyogova Eva, Zhou Haiyan, Maxwell Gillian K, Powis Rachael A, Muntoni Francesco, Gillingwater Thomas H, Parson Simon H

Supplementary Fig. 1 Molecular Regulatory Pathways are Modified in SMA Liver
Semi-quantitative RT-PCR analysis of *albumin* (A), *α-FTP* (B), *Ireb-2* (B) and *Annexin A2* (C) transcripts in control and SMA livers normalised to *Ppia* and *Oaz1*. (A) *Albumin* Control lanes = 10-12, *Albumin* SMA lanes = 13-15, *Ppia* Control lanes = 17-19, *Ppia* SMA lanes = 21-23, *Oaz1* Control lanes = 25-27, *Oaz1* SMA lanes = 29-31; (B) *α-FTP* Control lanes = 9-11, *α-FTP* SMA lanes = 13-15; *Ireb-2* Control lanes = 18-20, *Ireb-2* SMA lanes = 21-23, *Ppia* Control lanes = 33-35, *Ppia* SMA lanes = 37-39, *Oaz1* Control lanes = 41-43, *Oaz1* SMA lanes = 45-47; (C) *Annexin A2* Control lanes = 1-3, *Annexin A2* SMA lanes = 6-8. *Ppia* Control lanes = 9-11, *Ppia* SMA lanes = 13-15, *Oaz1* Control lanes = 17-19, *Oaz1* SMA lanes = 21-23. Total erythropoietin protein levels (D) analysed by Western Blot and normalised to the total protein (Instant Blue) (E).

Supplementary Fig. 2 Antisense Treatment Prolonged Life and Normalised Liver Development in SMA
Semi-quantitative RT-PCR of the *FL-SMN2 to Δ7 SMN2* transcripts in Control, SMA and antisense treated SMA liver at P11. Control lanes = 13,14,15 and 16; SMA lanes = 17,18,19 and 20; SMA+PMO25 = 21,22,23 and 24.

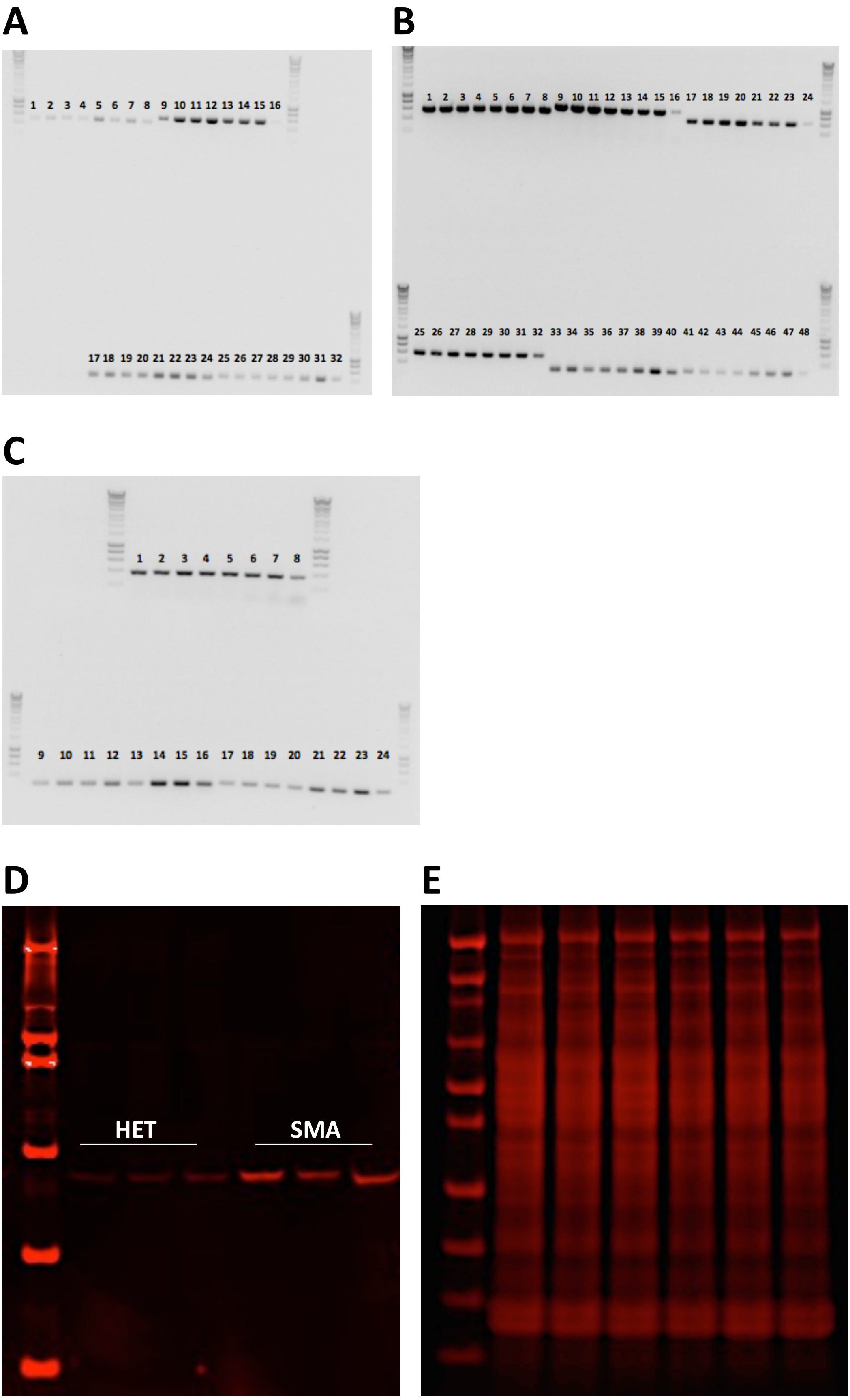

Supplementary Figure 1

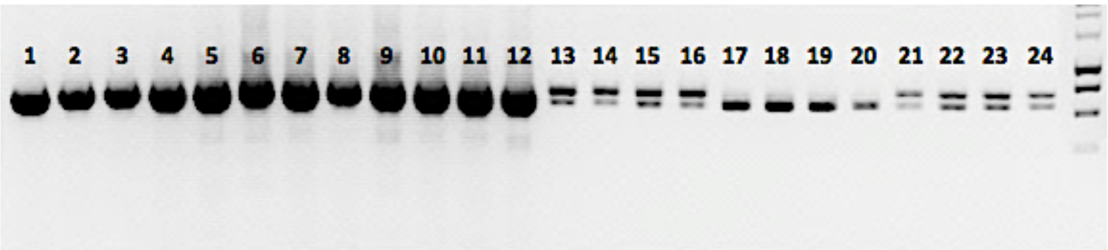

Supplementary Figure 2
